# Supplementary material for: Widespread somatic L1 retrotransposition in normal colorectal epithelium
Source: Nature. 2023 May 10;617(7961):540–7. doi: 10.1038/s41586-023-06046-z (PMC10191854; doi:10.1038/s41586-023-06046-z)
Supplement: Supplementary file 1 — This file contains Supplementary Discussion 1 (potential culture-associated L1 retrotransposition events in the clones), Supplementary Discussion 2 (genomics techniques for sensitive detection of soL1Rs), Supplementary Discussion 3 (panorama of promoter methylation and readthrough expression of rc-L1s), Supplementary References, Supplementary Figs. 1–5 and legends for Supplementary Tables 1–6. [file 41586_2023_6046_MOESM1_ESM.pdf]

nature portfolio

https://doi.org/10.1038/s41586-023-06046-z

Supplementary information

Widespread somatic L1 retrotransposition in normal colorectal epithelium

In the format provided by the authors and unedited

Supplementary Information for

Widespread somatic L1 retrotransposition in normal colorectal epithelium

Chang Hyun Nam, Jeonghwan Youk, Jeong Yeon Kim, Joonoh Lim, Jung Woo Park, Soo A Oh, Hyun Jung Lee, Ji Won Park, Hyein Won, Yunah Lee, Seung-Yong Jeong, Dong-Sung Lee, Ji Won Oh, Jinju Han, Junehawk Lee, Hyun Woo Kwon, Min Jung Kim, and Young Seok Ju

Table of contents

Supplementary Discussion 1. Potential culture-associated L1 retrotransposition events in the clones …...2

Supplementary Discussion 2. Genomics techniques for sensitive detection of soL1Rs …………………….2

Supplementary Discussion 3. Panorama of promoter methylation and read-through expression of rc-L1s…3

Supplementary Figures……………………………………………………………………………………...5

Supplementary Table Legends...…………………………………………………………………………...9

Supplementary Discussion 1. Potential culture-associated L1 retrotransposition events in the clones

Three lines of evidence indicated that most of the soL1Rs in the clones were true somatic events rather than culture-induced events. First, the VAFs for soL1Rs in clones were distributed approximately 50%, and thus soL1Rs were shared by all cells in a clone (**Supplementary Fig. 1a**). Similarly, in clones established from male donors, soL1Rs in non-pseudo-autosomal regions of chromosome X exhibited approximately 100% VAF. Second, as described in the main manuscript, the soL1R burden per clone increased with age, which was not possible if most instances were culture-mediated artifacts. Third, we experimentally confirmed the rate of culture-associated events in colorectal epithelium using 13 pairs of serial single-cell expansions, directly suggesting that >90% of the detected soL1Rs were true *in vivo* events (**Supplementary Fig. 1b**). In addition, the paucity of soL1Rs in the blood and fibroblast clones implies that cell culture conditions are not a sufficient condition for L1 retrotransposition.

Supplementary Discussion 2. Genomics techniques for sensitive detection of soL1Rs

For the detection of somatic mutations in single cells, several complementary techniques have been used[1]. In normal intestinal crypts containing thousands of cells, a few stem cells continuously compete with each other[2], resulting in single-clone compartments in the microscopic structure. As in cancer tissues, the cells in a crypt share all somatic mutations acquired in the genome of the founder stem cell. Using specialized technologies such as laser capture microdissection **(LCM)**, these patches can be physically separated, followed by whole-genome sequencing through an enzyme-based library preparation technique that allows a low input amount of genomic DNA[3]. In this way, somatic mutations in the normal colorectal epithelium, particularly single base substitutions and indels, were systematically detected and analyzed[4].

Although LCM is an efficient technique for exploring point mutations at single-cell resolution, we realized that this approach is not adequate for detecting somatic L1 retrotranspositions **(soL1Rs)**. As mentioned in the main manuscript, critical supporting evidence for soL1R detection includes the clustered reads carrying poly-A tails, which are imprints of reverse transcription of transcripts. However, these poly-A-carrying reads are technically depleted in the whole-genome sequences prepared through LCM-based technique[4] compared to those prepared through clonal expansions

(**Supplementary Figs. 2a-2c**). The poly-A dropout greatly reduces the sensitivity and precision of soL1R detection in current bioinformatics tools, i.e., MELT[5], TraFiC-mem[6], DELLY[7], and xTea[8].

The mechanism underlying the poly-A dropout is unclear. We speculate that it stems from the DNA preparation step: the enzymatic fragmentation, used by Lee-Six et al.[4], may have a bias or be less effective in fragmenting DNA in A (or T) homopolymeric regions **(Supplementary Fig. 3)**. Whichever the reason, we conclude that current LCM-based whole-genome sequencing datasets are practically not suitable for soL1R analysis until substantial improvements in the library preparation step, bioinformatics tools, or both.

Supplementary Discussion 3. Panorama of promoter methylation and read-through expression of rc-L1s

We analyzed whole-genome methylation sequencing and RNA sequencing on 132 colorectal and 7 fibroblast clones from 9 individuals to investigate the epigenetic regulation of L1 activity. Our analysis revealed a significant association between a decrease in L1 promoter methylation level and an increase in read-through expression **(Extended Data Fig. 6)**. Using the multi-omics datasets, we created a panorama of promoter methylation and read-through expression for the 156 retrotransposition-competent L1s **(rc-L1s)** present in the germline of the 9 individuals. The panorama includes 48 rc-L1 sources which contributed any transduction in the clones and/or whose promoters were substantially demethylated in five or more clones (**Extended Data Fig. 7**). The multi-omics panoramas of the other rc-L1 sources are shown in Supplementary Fig. 4 (sources with population allele frequency (PAF) ≥95%) and in Supplementary Fig. 5 (sources with PAF <95%).

References

1. Youk, J., Kwon, H. W., Kim, R. & Ju, Y. S. Dissecting single-cell genomes through the clonal organoid technique. *Exp Mol Med* **53**, 1503-1511, doi:10.1038/s12276-021-00680-1 (2021).
2. Barker, N. *et al.* Identification of stem cells in small intestine and colon by marker gene Lgr5. *Nature* **449**, 1003-1007, doi:10.1038/nature06196 (2007).
3. Ellis, P. *et al.* Reliable detection of somatic mutations in solid tissues by laser-capture microdissection and low-input DNA sequencing. *Nature Protocols* **16**, 841-871, doi:10.1038/s41596-020-00437-6 (2021).
4. Lee-Six, H. *et al.* The landscape of somatic mutation in normal colorectal epithelial cells. *Nature* **574**, 532-537, doi:10.1038/s41586-019-1672-7 (2019).
5. Gardner, E. J. *et al.* The Mobile Element Locator Tool (MELT): population-scale mobile element discovery and biology. *Genome Res.* **27**, 1916-1929, doi:10.1101/gr.218032.116 (2017).
6. Tubio, J. M. C. *et al.* Mobile DNA in cancer. Extensive transduction of nonrepetitive DNA mediated by L1 retrotransposition in cancer genomes. *Science* **345**, 1251343, doi:10.1126/science.1251343 (2014).
7. Rausch, T. *et al.* DELLY: structural variant discovery by integrated paired-end and split-read analysis. *Bioinformatics* **28**, i333-i339, doi:10.1093/bioinformatics/bts378 (2012).
8. Chu, C. *et al.* Comprehensive identification of transposable element insertions using multiple sequencing technologies. *Nat. Commun.* **12**, 3836, doi:10.1038/s41467-021-24041-8 (2021).

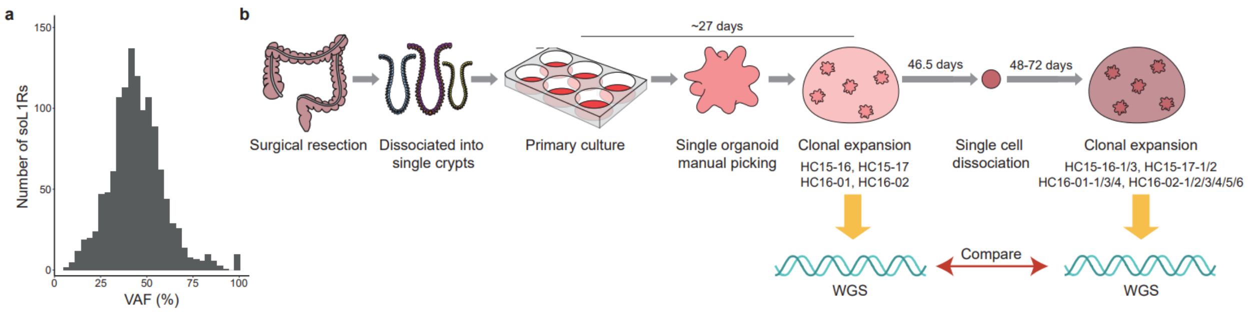

Supplementary Fig. 1. Evidences supporting that most of the soL1Rs were true somatic events.

a, The distribution of VAFs of 1,236 soL1Rs identified in normal colorectal clones. A peak VAF near 0.5 suggests that the majority of soL1Rs detected were shared by all cells in a clone, therefore unlikely to be culture-associated events. **b,** Experimental design for estimating the rate of L1 retrotransposition during culture. For whole-genome sequencing of clones, a single crypt from the surgical specimen (naturally clonalized) was cultured for 27 days. The second clonalization was conducted after additional culture of 46.5 days on average (ranging between 43-50d). From 13 pairs of whole-genome sequences of early and late clones, we found ten new clonal soL1R events specifically in late clones, which should be acquired during cell culture before the second clonalization, or ~73.5 days (27d+46.5d). This allowed us to calculate the culture-associated soL1R rate: 10 soL1Rs / 13 clones / 73.5d = 0.01 per clone per day. Using the rate, we can estimate the upper boundary of the rate, which is 0.01 per clone per day X 27 days = 0.28 soL1Rs, assuming the expansion of the most recent common ancestral cell (MRCA) at day 27. Of note, the lower boundary is 0 if the expansion of the MRCA occurred at day 0. It suggests that the proportion of the culture-associated soL1Rs is 9% at maximum, which is 0.28/3.044 X 100 (3.044 soL1Rs detected per clone, 1,236 soL1Rs from 406 clones).

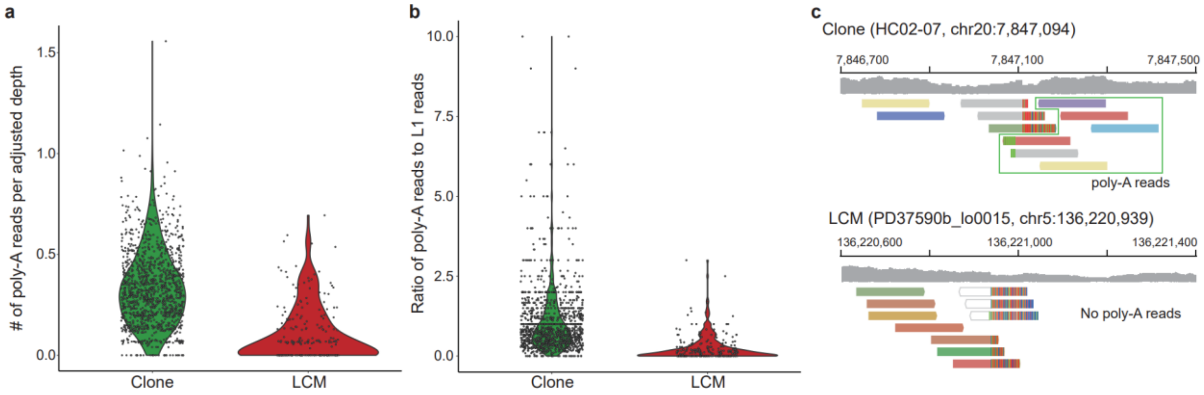

Supplementary Fig. 2. Lack of poly-A reads in whole-genome sequences prepared using LCM.

a, Number of poly-A reads per adjusted depth. The soL1Rs detected in whole-genome sequences prepared using LCM have fewer poly-A reads per depth compared to those from clonal expansion (0.33 vs. 0.11 on average for clonal expansion and LCM, respectively, P=3.1×10^{-59}, two-sided t-test). Poly-A reads indicate the read pairs with poly-A sequences in the reads or their mate reads. Sequencing depth was adjusted with purity, which is estimated as two times peak VAF. soL1R, somatic L1 retrotransposition; LCM, laser capture microdissection; VAF, variant allele fraction. **b**, Ratio of poly-A reads to L1 reads. The ratio is lower in soL1Rs detected in whole-genome sequences prepared from LCM than those from clonal expansion (1.07 vs. 0.26 on average for clonal expansion and LCM, respectively, P=2.2×10^{-72}, two-sided t-test). L1 reads indicate the read pairs with L1 consensus sequences in the reads or their mate reads. **c**, Examples of soL1Rs detected in whole-genome sequences prepared using clonal expansion (top) and LCM (down).

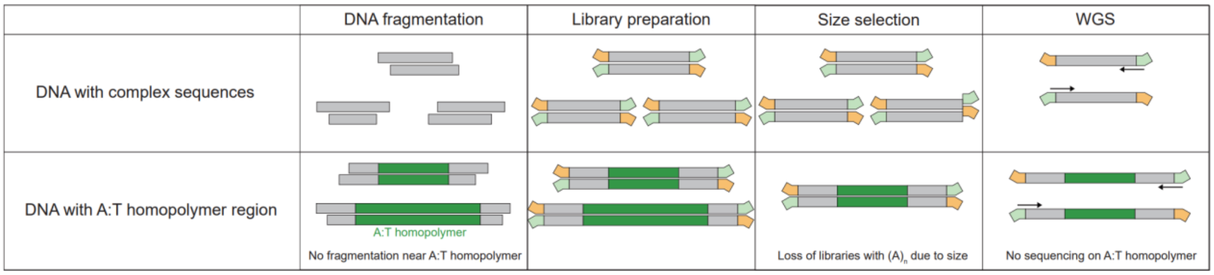

Supplementary Fig. 3. Proposed mechanism underlying poly-A dropout in whole-genome sequences prepared using LCM.

If DNA with A:T homopolymer regions are less vulnerable to enzymatic fragmentation, A:T homopolymer regions are likely to be included in large-sized libraries, which could be eliminated during the size selection process. Even if A:T homopolymer containing libraries survive, A:T homopolymer region far from the adapter at both ends would not be sequenced. LCM, laser capture microdissection.

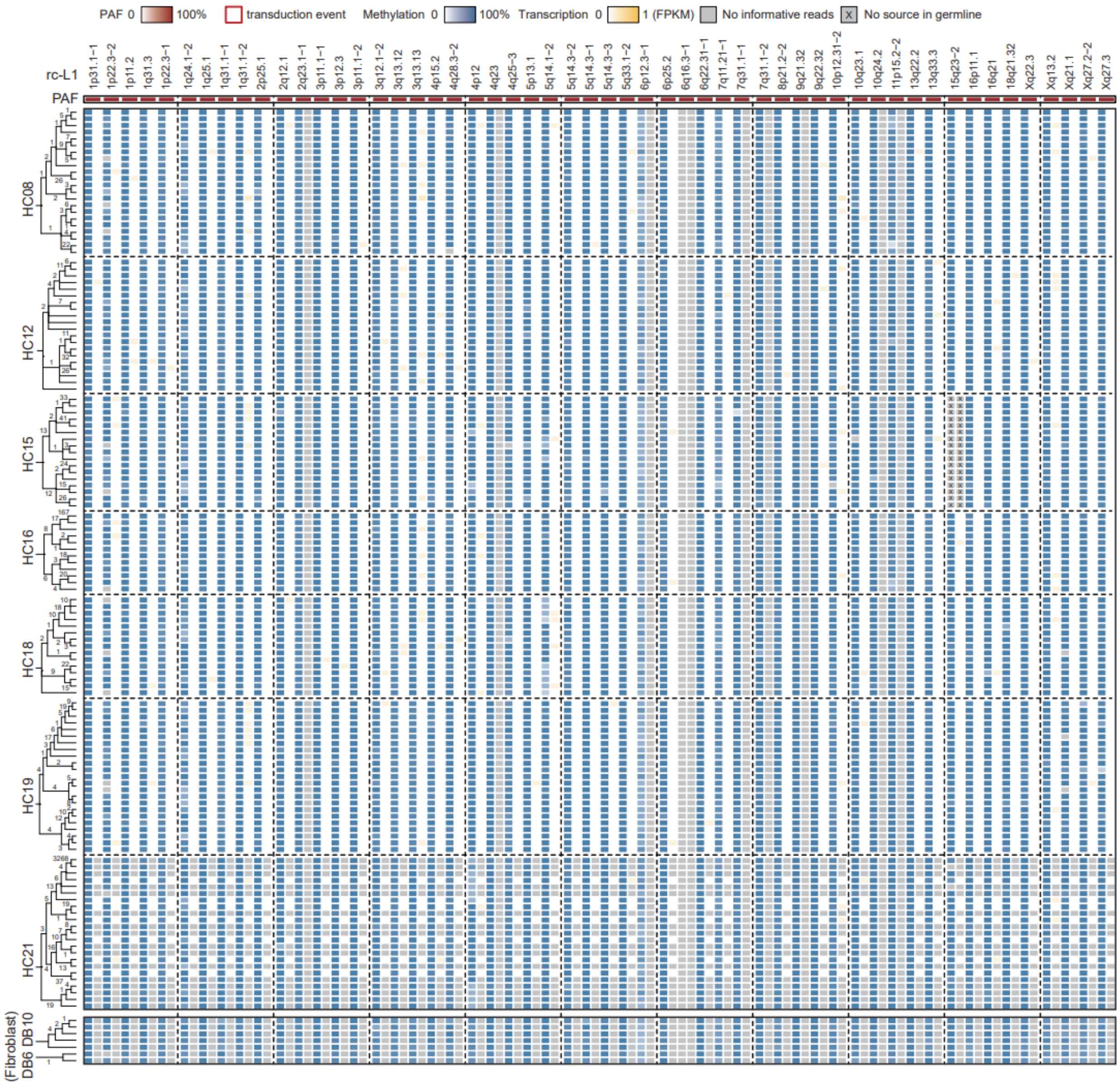

Supplementary Fig. 4. Panorama of DNA methylation status and read-through RNA expression levels of 54 rc-L1s with PAF greater than 95%.

DNA methylation status, read-through RNA expression levels, and developmental phylogenies of 54 rc-L1s that were inactive, generally had closed promoters in our colorectal cohort, and showed PAF greater than 95% are displayed. The phylogenies are shown on the left side with the number of point mutations (molecular time). PAF, population allele frequency; FPKM, fragments per kilobase transcript per million; rc-L1, retrotransposition-competent L1.

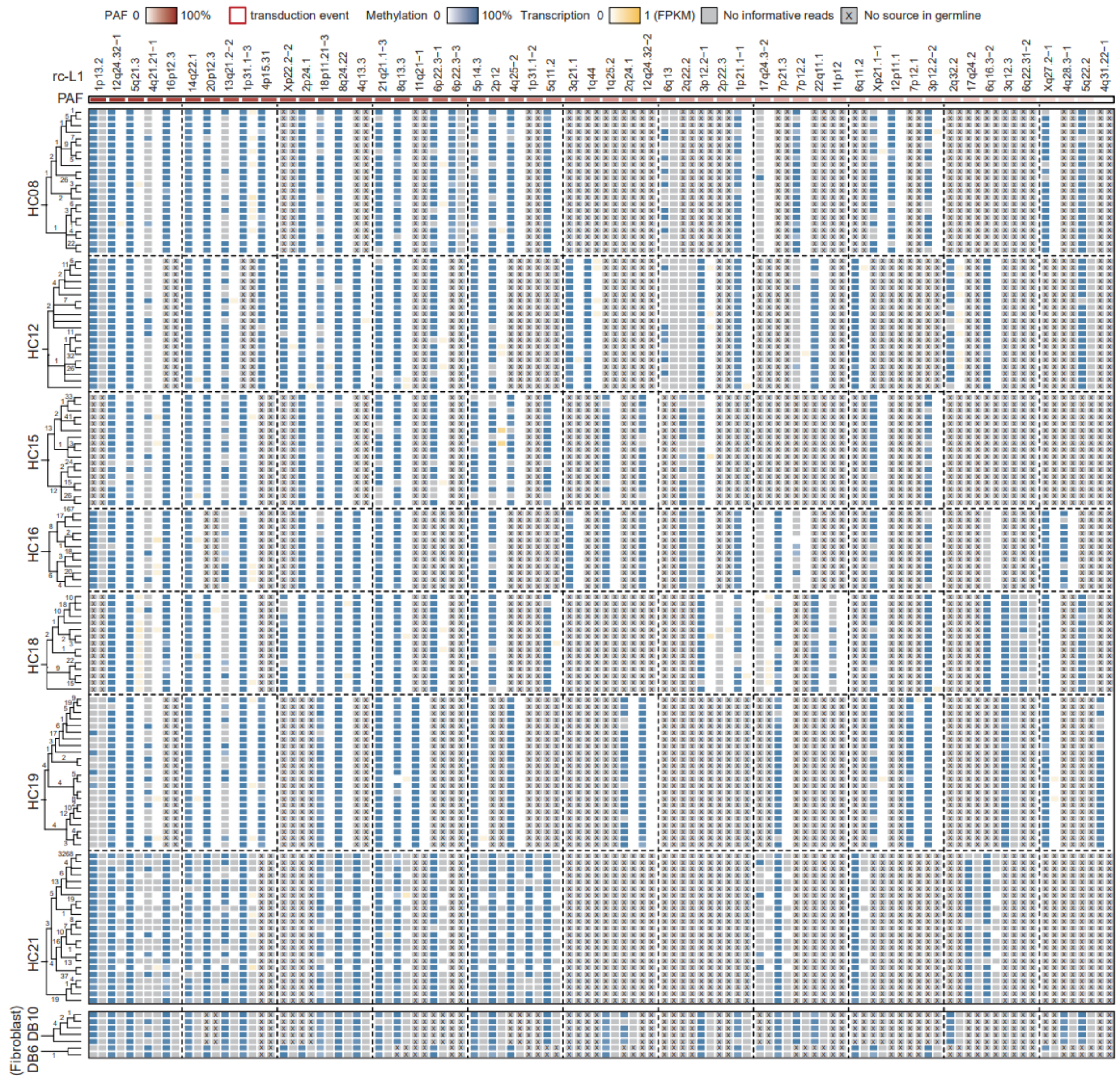

Supplementary Fig. 5. Panorama of DNA methylation status and read-through RNA expression levels of 54 rc-L1s with PAF less than 95%.

DNA methylation status, read-through RNA expression levels, and developmental phylogenies of 54 rc-L1s that were inactive, generally had closed promoters in our colorectal cohort, and showed PAF less than 95% are displayed. The phylogenies are shown on the left side with the number of point mutations (molecular time). PAF, population allele frequency; FPKM, fragments per kilobase transcript per million; rc-L1, retrotransposition-competent L1.

Supplementary Table Legend

Supplementary Table 1. Demographic and mutational characteristics of samples. The table presents information on each sample in the study, including the patient's age and sex, the anatomical location where the samples was obtained, as well as the mutational burden and signatures for each sample.

Supplementary Table 2. Annotation of somatic retrotranspositions identified in this study. The table presents detailed information on each somatic retrotransposition events identified in the study, including the genomic coordinates, strand orientation, insertion size, type, and the number of supporting reads for each insertion.

Supplementary Table 3. Annotation of somatic retrotranspositions identified in LCM-based patches. The table presents detailed information on each somatic retrotransposition events identified in LCM-based patches, including the genomic coordinates, strand orientation, insertion size, type, and the number of supporting reads for each insertion.

Supplementary Table 4. List of source elements with characteristics. The table contains information on 276 rc-L1s analysed in the study, including genomic coordinates, cytoband, strand orientation, population allele frequency, L1 subfamily information, and a list of truncating mutations. Additionally, the table shows the number of transduction events from each rc-L1 in every colorectal sample examined in the study.

Supplementary Table 5. Association of soL1R and genome instability features in cancer. The table presents information on colorectal cancer samples analysed in the study and PCAWG whitelisted cancers, including histology information, the number of somatic L1 retrotransposition and structural variations, whether the samples harbour TP53 inactivating mutations or microsatellite instability, and a list of canonical driver mutations.

Supplementary Table 6. Organoid culture media composition for colorectal epithelial cells. This table presents organoid culture media composition for colorectal epithelial cells.
