# Supplementary material for: Widespread somatic L1 retrotransposition in normal colorectal epithelium
Source: Nature. 2023 May 10;617(7961):540–7. doi: 10.1038/s41586-023-06046-z (PMC10191854; doi:10.1038/s41586-023-06046-z)
Supplement: Supplementary file 2 — Reporting Summary [file 41586_2023_6046_MOESM2_ESM.pdf]

Corresponding author(s): Hyun Woo Kwon, Min Jung Kim, and Young Seok Ju

Last updated by author(s): Mar 22, 2023

# Reporting Summary

Nature Portfolio wishes to improve the reproducibility of the work that we publish. This form provides structure for consistency and transparency in reporting. For further information on Nature Portfolio policies, see our Editorial Policies and the Editorial Policy Checklist.

## Statistics

For all statistical analyses, confirm that the following items are present in the figure legend, table legend, main text, or Methods section.

| n/a | Confirmed | |
|---|---|---|
| ☐ | ☒ | The exact sample size (*n*) for each experimental group/condition, given as a discrete number and unit of measurement |
| ☐ | ☒ | A statement on whether measurements were taken from distinct samples or whether the same sample was measured repeatedly |
| ☐ | ☒ | The statistical test(s) used AND whether they are one- or two-sided<br>*Only common tests should be described solely by name; describe more complex techniques in the Methods section.* |
| ☐ | ☒ | A description of all covariates tested |
| ☐ | ☒ | A description of any assumptions or corrections, such as tests of normality and adjustment for multiple comparisons |
| ☐ | ☒ | A full description of the statistical parameters including central tendency (e.g. means) or other basic estimates (e.g. regression coefficient) AND variation (e.g. standard deviation) or associated estimates of uncertainty (e.g. confidence intervals) |
| ☐ | ☒ | For null hypothesis testing, the test statistic (e.g. *F*, *t*, *r*) with confidence intervals, effect sizes, degrees of freedom and *P* value noted<br>*Give P values as exact values whenever suitable.* |
| ☒ | ☐ | For Bayesian analysis, information on the choice of priors and Markov chain Monte Carlo settings |
| ☒ | ☐ | For hierarchical and complex designs, identification of the appropriate level for tests and full reporting of outcomes |
| ☒ | ☐ | Estimates of effect sizes (e.g. Cohen's *d*, Pearson's *r*), indicating how they were calculated |

*Our web collection on statistics for biologists contains articles on many of the points above.*

## Software and code

Policy information about availability of computer code

| | |
|---|---|
| Data collection | No software was used. |
| Data analysis | We aligned whole-genome sequencing reads to the human reference genome (GRCh37) using BWA(0.7.17) algorithm. The duplicated reads were removed by Picard (2.1.0) or SAMBLASTER(0.1.24). We identified single-nucleotide variants and short indels using Varscan2(2.4.2) and HaplotyperCaller2 in GATK(4.0.0.0). In addition, we identified somatic genomic rearrangements using DELLY(0.7.6) and called somatic L1 retrotransposition using MELT(2.2.0), TraFiC-mem(1.2.0), xTea(0.1), as well as DELLY(0.7.6). Detected variants were inspected using IGV(2.8.2). Long-read sequences generated by the PacBio platform were aligned to human reference genome (GRCh37) using pbmm2(1.4.0). We removed the adaptor sequences from RNAseq and EM-seq reads using Cutadapt(1.18) and aligned to the human reference genome (GRCh37) using BWA(0.7.17) and Bismark(0.22.3), respectively. Signature analysis was performed using SigProfiler(1.1.4) and HDP(0.1.5). Custom scripts were written by Python(3.6.10, 2.7) and R(3.6.0) and are available at Github (https://github.com/ju-lab/colon_LINE1). |

For manuscripts utilizing custom algorithms or software that are central to the research but not yet described in published literature, software must be made available to editors and reviewers. We strongly encourage code deposition in a community repository (e.g. GitHub). See the Nature Portfolio guidelines for submitting code & software for further information.

## Data

Policy information about availability of data

All manuscripts must include a data availability statement. This statement should provide the following information, where applicable:

- Accession codes, unique identifiers, or web links for publicly available datasets
- A description of any restrictions on data availability
- For clinical datasets or third party data, please ensure that the statement adheres to our policy

Whole-genome, DNA methylation, and transcriptome sequencing data are deposited in the European Genome-phenome Archive (EGA) with accession EGAS00001006213 and available for general research use. Human reference genome (GRCh37) is available at NIH websites (https://www.ncbi.nlm.nih.gov/data-hub/genome/GCF_000001405.13).

## Human research participants

Policy information about studies involving human research participants and Sex and Gender in Research.

| | |
|---|---|
| Reporting on sex and gender | Sex information of the study participants were initially provided from hospital and confirmed by sequencing depth of sex chromosomes in whole-genome sequencing. The information is available in Supp Table 1. We did not find any differences in L1 activity between males and females. Sex and gender were not considered in study design. |
| Population characteristics | Out of 28 patients, 20 were diagnosed with colorectal cancer. The age of the patients spanned several age groups ranging from 37 to 93. The ratio between males and famales were almost 1:1 (13 males and 15 females). |
| Recruitment | We recruited participants from those who planned to undergo colectomy surgery (n=19) or colonoscopic colon polypectomy (n=1) in Seoul National University Hospital. Participants were identified through a review of medical records as were approached in the clinic to obtain informed consent. There is a potential bias since all recruited participants had a diagnosis of colorectal disease. Data for the other individuals (7 for fibroblast and 1 for blood clones) were published previously and downloaded for this study. |
| Ethics oversight | All the procedures in this study were approved by the Institutional Review Board of Seoul National University Hospital (approval number: 1911-106-1080) and Korea Advanced Institute of Science and Technology (approval number: KH2022-058). |

Note that full information on the approval of the study protocol must also be provided in the manuscript.

# Field-specific reporting

Please select the one below that is the best fit for your research. If you are not sure, read the appropriate sections before making your selection.

☒ Life sciences ☐ Behavioural & social sciences ☐ Ecological, evolutionary & environmental sciences

For a reference copy of the document with all sections, see nature.com/documents/nr-reporting-summary-flat.pdf

# Life sciences study design

All studies must disclose on these points even when the disclosure is negative.

| | |
|---|---|
| Sample size | No statistical methods were used to predetermine sample size. We selected samples from available individuals to describe the mutational landscape of LINE1 retrotransposition in normal cells. |
| Data exclusions | No data were excluded from the analyses. |
| Replication | We repeated the colon organoid culture and generated 13 pairs of mother-daughter colon organoids. The main purpose was to estimate the rate of culture-associated L1s, but all the somatic L1 retrotransposition events identified in mother organoids were validated in daughter organoids. |
| Randomization | We did not perform randomization because this study did not involve experimental groups. Covariates were controlled by statistical methods. |
| Blinding | Blinding is not applicable because this is a descriptive study. |

# Reporting for specific materials, systems and methods

We require information from authors about some types of materials, experimental systems and methods used in many studies. Here, indicate whether each material, system or method listed is relevant to your study. If you are not sure if a list item applies to your research, read the appropriate section before selecting a response.

## Materials & experimental systems

| n/a | Involved in the study |
|---|---|
| ☒ | ☐ Antibodies |
| ☒ | ☐ Eukaryotic cell lines |
| ☒ | ☐ Palaeontology and archaeology |
| ☒ | ☐ Animals and other organisms |
| ☒ | ☐ Clinical data |
| ☒ | ☐ Dual use research of concern |

## Methods

| n/a | Involved in the study |
|---|---|
| ☒ | ☐ ChIP-seq |
| ☒ | ☐ Flow cytometry |
| ☒ | ☐ MRI-based neuroimaging |
